# Six practical recommendations for improved implementation outcomes reporting

**DOI:** 10.1186/s13012-021-01183-3

**Published:** 2022-02-08

**Authors:** Rebecca Lengnick-Hall, Donald R. Gerke, Enola K. Proctor, Alicia C. Bunger, Rebecca J. Phillips, Jared K. Martin, Julia C. Swanson

**Affiliations:** 1 The Brown School, Washington University in St. Louis, St. Louis, USA; 2 Graduate School of Social Work, University of Denver, Denver, CO USA; 3 Shanti Khinduka Distinguished Professor Emerita, The Brown School, Washington University in St. Louis, St. Louis, USA; 4 College of Social Work, The Ohio State University, Columbus, OH USA; 5 College of Education & Human Ecology, The Ohio State University, Columbus, OH USA

**Keywords:** Acceptability, Adoption, Appropriateness, Feasibility, Fidelity, Implementation cost, Penetration, Sustainability, Implementation outcome

## Abstract

**Background:**

Implementation outcomes research spans an exciting mix of fields, disciplines, and geographical space. Although the number of studies that cite the 2011 taxonomy has expanded considerably, the problem of harmony in describing outcomes persists. This paper revisits that problem by focusing on the clarity of reporting outcomes in studies that examine them. Published recommendations for improved reporting and specification have proven to be an important step in enhancing the rigor of implementation research. We articulate reporting problems in the current implementation outcomes literature and describe six practical recommendations that address them.

**Recommendations:**

Our first recommendation is to clearly state each implementation outcome and provide a definition that the study will consistently use. This includes providing an explanation if using the taxonomy in a new way or merging terms. Our second recommendation is to specify how each implementation outcome will be analyzed relative to other constructs. Our third recommendation is to specify “the thing” that each implementation outcome will be measured in relation to. This is especially important if you are concurrently studying interventions and strategies, or if you are studying interventions and strategies that have multiple components. Our fourth recommendation is to report who will provide data and the level at which data will be collected for each implementation outcome, and to report what kind of data will be collected and used to assess each implementation outcome. Our fifth recommendation is to state the number of time points and frequency at which each outcome will be measured. Our sixth recommendation is to state the unit of observation and the level of analysis for each implementation outcome.

**Conclusion:**

This paper advances implementation outcomes research in two ways. First, we illustrate elements of the 2011 research agenda with concrete examples drawn from a wide swath of current literature. Second, we provide six pragmatic recommendations for improved reporting. These recommendations are accompanied by an audit worksheet and a list of exemplar articles that researchers can use when designing, conducting, and assessing implementation outcomes studies.

**Supplementary Information:**

The online version contains supplementary material available at 10.1186/s13012-021-01183-3.

Contributions to the literature

- Rigorous and consistent reporting of implementation outcomes is necessary for synthesizing insights about the effectiveness of implementation strategies across studies conducted in diverse contexts.
- It is also necessary for building strong theory about implementation mechanisms.
- This manuscript describes common reporting problems identified in the implementation outcomes literature.
- We offer six recommendations for improving implementation outcome reporting. We also provide two tools (an audit worksheet and a list of exemplar articles) to help readers put these recommendations into practice.

## Background

Implementation researchers study the process of transitioning evidence-based interventions from controlled research environments to real-world practice settings [1]. A primary focus of implementation science is the study of implementation outcomes, defined as “the effects of deliberate and purposive actions to implement new interventions” [2]. Implementation outcomes are used to evaluate implementation success and processes and are often employed as intermediate outcomes in studies of intervention effectiveness or quality [3]. To clarify and standardize terminology and to promote increased rigor in implementation science, a 2011 paper [3] put forward a research agenda and a taxonomy of eight discrete implementation outcomes: acceptability, adoption, appropriateness, feasibility, fidelity, implementation cost, penetration, and sustainability.

Acceptability is the perception among stakeholders that a given treatment, service, practice, or innovation is agreeable, palatable, or satisfactory [3]. Adoption is the intent, initial decision, or action to try or employ an innovation or evidence-based practice; also referred to as uptake [3]. Appropriateness is the perceived fit, relevance, or compatibility of an innovation or evidence-based practice for a given practice setting, provider, or consumer; and/or perceived fit of the innovation to address a particular issue or problem [3]. Feasibility is the extent to which a new treatment or an innovation can be successfully used or carried out in a given setting [3]. Fidelity is the degree to which an intervention was implemented as it was prescribed in the original protocol or as was intended by program developers [3]. Implementation cost is the cost impact of an implementation effort [3]. Penetration is the integration or saturation of an intervention within a service setting and its subsystems; calculated as a ratio of those to whom the intervention is delivered divided by the number of eligible or potential recipients [3]. Last, sustainability is the extent to which a newly implemented treatment is maintained or institutionalized within a service setting’s ongoing, stable operations [3].

Implementation outcomes research spans an exciting mix of fields, disciplines, and geographical space. In the last 10 years, the 2011 paper was cited over 1600 times in Web of Science. Implementation outcomes research can be found in primary and hospital care [4, 5], behavioral health [6], child welfare and parenting [7, 8], HIV prevention and care [9, 10], school-based services [11, 12], and other settings. Moreover, the taxonomy proposed in the 2011 paper is included in funding announcements that guide implementation research design. One such example is the US National Institute of Health’s PAR-19-276 Dissemination and Implementation Research in Health [13], which has 17 participating institutes and specifies that the inclusion of implementation outcomes is an important component to include in funding proposals. Though the number of studies that cite the 2011 taxonomy has expanded considerably, the problem of harmony in describing outcomes persists. This paper revisits that problem by focusing on the clarity of reporting outcomes in studies that examine them.

Published recommendations for improved reporting and specification have proven to be an important step in enhancing the rigor of implementation research. The Standards for Reporting Implementation Studies (StaRI), for example, was developed in 2017 and includes 27 checklist items that disentangle the implementation strategy from the intervention [14, 15]. The StaRI is intentionally open to the range of research designs and methods that implementation researchers use [14, 15]. This contrasts with more narrowly focused reporting guidelines, like the Consolidated Standards of Reporting Trials for randomized control trials [16–18] and the Standard Protocol Items: Recommendations for Interventional Trials for clinical trial protocols [19]. Other reporting guidelines used in implementation research focus on “the thing” [20] being implemented. Examples include the Workgroup for Intervention Development and Evaluation Research recommendations [21, 22] and the Template for Intervention Description and Replication checklist [23]. As Rudd and colleagues aptly explained, reporting guidelines are important because they improve the rigor and value of implementation research by supporting replication, research synthesis, and dissemination; this increases the speed at which practitioners can use empirical findings [24].

### Paper goals

As part of a scoping review aimed at describing how the field conceptualized, measured, and advanced theory around implementation outcomes since 2011 [25], we identified 358 empirical studies published in peer-reviewed journals that cited the original paper and assessed one or more implementation outcomes. We found that while anchored by the conceptual definitions from the 2011 taxonomy, these studies varied substantially in how implementation outcomes were reported. This is a problem because variations limit the synthesis of knowledge and theory generation across studies, interventions, and settings. This also echoes concerns raised in the implementation outcomes research agenda set forth in 2011 [3]. The aims of this paper are to articulate reporting problems in the current implementation outcomes literature and describe six practical recommendations that address them. We also present an audit worksheet that readers can use to plan and assess their own work and a list of exemplar articles that readers can use as a reference. We hope that these tools can help build capacity among implementation researchers, and support reviewers and editors who are positioned to offer constructive feedback to grant writers and authors in service of advancing a harmonized literature on implementation outcomes.

### How we identified reporting problems in the implementation outcomes literature

To identify current reporting problems and generate meaningful recommendations, we leveraged elements of the data charting stage of our scoping review methodology [25]. During data charting, team members trained in implementation research reviewed full-text articles to identify the implementation outcomes examined, measurement approaches, and other study details. However, when these study features were too unclear for basic data charting as this stage got underway, team members brought these issues to the whole team to discuss. This process led to significant refinements in the data charting form, and stronger consensus within the team about how to chart these studies considering the wide outcomes reporting variations. Yet, the team continued to encounter data charting difficulties and members requested additional consultation from the protocol authors for about 8% of the articles reviewed. This reflected a substantial number of articles with reporting issues that went beyond the expert team’s consensus building discussions and data charting form iterations. Most of the challenges discussed in the one-on-one consultation meetings via email or video conference (which were documented) were related to insufficient detail reported about the implementation outcomes.

To synthesize these reporting issues systematically across the coders, we held weekly meetings among protocol authors and conducted a full team meeting during the data charting process (after charting about 250 articles). During the weekly protocol author meetings, we discussed consultation issues and decisions that were made during the past week and began to identify themes and patterns related to reporting problems that were emerging across studies and team members. During our full team meeting, the first author presented an initial list of reporting problems and asked each person (*n* = 7) to elaborate, add to the list with their own examples, and reflect upon the list’s alignment with their own coding experience. Detailed meeting notes and the Zoom chat transcript were retained to inform these recommendations.

## Current problems and recommendations for improvement

We next present our list of six identified reporting problems and proposed recommendations to prevent them in future work. To accompany this, we created two tools. Additional file 1 is an audit worksheet that the reader can use to assess adherence to our proposed recommendations or to plan out the inclusion of implementation outcomes in potential work. In Additional file 2, we provide exemplar articles that the reader may use as a guide and to generate ideas.

### Recommendation 1: consistent term use

The 2011 paper noted widespread inconsistency in terminology for implementation outcomes and called for consistent use of language in describing key constructs [3]. Our review revealed that this problem prevails and can appear in the literature in three specific ways. One way was reporting different outcomes in different manuscript sections. In one article, for example, the stated study goal in the Introduction was to assess fidelity and sustainment. However, the authors only reported on fidelity in the “Methods” and “Results” section, never addressing results pertaining to sustainment. Whether these authors failed to distinguish between fidelity and sustainment conceptually and operationally, or whether the paper simply failed to address sustainment, the effect is the same: lack of clarity about the specific outcome being addressed. Inconsistent terminology prevents readers from knowing what construct was assessed and what exactly was learned—both of which prevent the accrual of information across studies.

Another way that this problem appeared was using terms from the 2011 taxonomy in a new way and without explanation. While the original taxonomy invited the identification and study of additional implementation outcomes, interchanged use of terms perpetuates confusion and impedes synthesis across studies [16]. Examples included an article where authors reported that they were assessing fidelity but called it uptake and an article where the definition of feasibility included the term acceptability. In both cases, an explanation as to why the outcome terms were applied in this way (while still citing the 2011 taxonomy) was absent.

Third, we found confusing instances of studies that merged implementation outcomes in the analysis and interpretation of results without explanation. For example, in one article, fidelity and acceptability were combined and called feasibility. In another, acceptability, feasibility, and appropriateness were combined into a variable called value. The 2011 research agenda described how implementation outcomes could be used in a “portfolio” of factors that explain implementation success [3]. For example, implementation success could be conceptualized as a combination of treatment effectiveness, acceptability, and sustainability [3]. However, understanding the role of implementation outcomes in mechanisms of change—including how we get to “implementation success” and what it looks like—requires precision in outcomes measurement and reporting. Until we have a stronger knowledge base, our field needs concepts to be disentangled rather than merged, absent compelling theory or evidence for combining. To address these reporting problems, *our first recommendation is to clearly state each implementation outcome and provide an operational definition that the study will use. Ensure consistent use of outcomes terms and operational definitions across manuscript sections and provide an explanation if using the taxonomy in a new way or merging terms.*

### Recommendation 2: role in analysis

Another reporting problem is lack of specificity around how the outcome was measured relative to other constructs. This problem appeared as poor or unclear alignment between outcomes-related aims, research questions and/or hypotheses, and the reported results. One example of this was an article that aimed to examine fidelity, adoption, and cost across multiple phases of implementation. However, the authors assessed barriers to adoption instead of actual adoption and used the terms fidelity, engagement, and adoption interchangeably when reporting results on the intervention and implementation strategies. This made it difficult to assess the roles that different implementation outcomes played in the study. In another article, the authors stated that their qualitative interview guide “provided insight into” acceptability, adoption, and appropriateness of the practice of interest. However, the “Results” section did not include any information about these implementation outcomes, and they were not mentioned again until the discussion of future directions.

*Our second recommendation is to specify how each implementation outcome will be or was analyzed relative to other constructs.* Readers can draw upon the categories that we observed during data charting. For example, an implementation outcome may be treated as an independent, dependent, mediating, moderating, or descriptive variable. Correlations may be assessed between an implementation outcome and another implementation outcome or a contextual variable. An implementation outcome may be treated as a predictor of system or clinical outcomes, or as an outcome of a planned implementation strategy. Manuscripts that succinctly list research questions or study aims—detailing the outcome variables measured and their role in analyses—are easier to identify in literature searches, easier to digest, and contribute to the accrual of information about the attainment and effects of specific implementation outcomes.

### Recommendation 3: referent

The next problem that we observed is difficulty identifying what “thing” [20] the implementation outcome is referring to. For example, in one article that examined both an intervention and an implementation strategy, the aims referred to feasibility and acceptability of the intervention. However, the “Results” section only reported on intervention acceptability and the “Discussion” section mentioned that acceptability and feasibility of the intervention using the implementation strategy were assessed. This example illustrates how study conclusions can be confusing when the implementation outcome referent is unclear. Another study compared different training approaches for promoting fidelity within a process improvement study. However, we were unable to discern whether fidelity was referring to the process improvement model, the training approaches, or both. As a result, it was difficult to assess which body of fidelity literature these findings pertained to.

As such,* our third recommendation is to specify “the thing” *[20] *that each implementation outcome will be measured in relation to*. This requires a thorough review of all manuscript sections and can be especially important if you are concurrently studying interventions and strategies (e.g., in a hybrid study [26]), or if you are studying interventions and strategies that have multiple components of interest. Coding options for “the thing” in our scoping review included screening, assessment, or diagnostic procedures (e.g., X-rays), one manualized treatment, program, or intervention (e.g., trauma-focused cognitive behavioral therapy), or multiple manualized interventions that are simultaneously implemented. We also observed that “the thing” may refer to research evidence or guidelines. It could be an administrative intervention (e.g., billing system, care coordination strategy, supervision approach), a policy, technology (e.g., health information technology, health app, data system), a form of outcome monitoring (e.g., measurement-based care for individual clients), data systems, indicators, or monitoring systems. Finally, “the thing” that an outcome is being measured in relation to may be a clinical pathway or service cascade intervention (e.g., screening, referral, treatment type of program).

### Recommendation 4: data source and measurement

The fourth reporting problem is lack of detail around how the implementation outcome was measured, including what data were used. For instance, some studies drew upon participant recruitment and retention information to reflect feasibility without describing the way this information was obtained or recorded. The “Methods” section of another article stated that “project records” were used to assess fidelity without providing additional detail. Another example is an article in which the “Measures” section stated that survey items were created by the study team based on the 2011 taxonomy, including feasibility, acceptability, and sustainability. However, appropriateness was the only one that was clearly operationalized in the “Methods” section. Lack of information about data source and measurement limits transparency, the ability to understand the strengths and limitations of different measurement approaches for implementation outcomes, and replication.

To address this, our fourth recommendation is twofold. *The first element of our recommendation is to report who provided data and the level at which data were collected for each implementation outcome*. The reader can consider the following categories when reporting this information for their studies. Possible options for who reported the data for an implementation outcome include client/patient, individual provider, supervisor/middle manager, administrator/executive leader, policymaker, or another external partner. Possible options for the level at which data were collected include individual/self, team/peers, organization, or larger system environment and community. We found that implementation outcomes studies often drew upon multiple levels of data (see also “Recommendation 6: unit of analysis vs. unit of observation”). Furthermore, the level at which data were collected and the level at which data were reported may not be the same (e.g., individual providers reporting on an organizational level implementation outcome variable). To address this, *the second element of our recommendation is to report what type of data was or will be collected and used to assess each implementation outcome*. Data may be quantitative, qualitative, or both. Information may be collected from interviews, administrative data, observation, focus groups, checklists, self-reports, case audits, chart or electronic health record reviews, client reports, responses to vignettes, or a validated survey instrument or questionnaire.

### Recommendation 5: timing and frequency

Another reporting problem that we encountered is lack of information about the timing and frequency of implementation outcome measurement. A fundamental principle of clear research reporting includes disclosing observation periods, times between observations, and number of times constructs are measured. Yet, our review of implementation outcome research was hampered by lack of such details. For example, in one article, self-assessments and independent assessments of fidelity were compared for a particular intervention. However, in the “Methods” section, fidelity assessments of both types were described as “completed during the last quarter” of a particular year. Without further detail, it was difficult to tell if these were cross-sectional fidelity assessments for unique providers or longitudinal data that tracked the same provider’s fidelity over time. Lack of detail about data collection timeframes limits researchers’ ability to assess the internal validity of study findings and the actual time that it takes to observe change in a given implementation outcome (and at a particular level of analysis). Therefore, *our fifth recommendation is to state the number of time points and the frequency at which each outcome was or will be measured*. Broad categories that the reader may consider include measuring the implementation outcome once (cross-sectional), twice (pre-post), or longitudinally (three or more time points are assessed). Reporting the phase [27] or stage [28] can also help to clarify when during the implementation lifecycle outcomes are observed or are most salient.

### Recommendation 6: unit of analysis vs. unit of observation

The last problem we encountered is inconsistent or insufficient specification of the unit of analysis (the unit for which we make inferences about implementation outcomes) and the unit of observation (most basic unit observed to measure the implementation outcome). In multiple instances, studies relied on reports from individual providers or clinicians to make inferences about team or organizational implementation outcomes (e.g., aggregating observations about individual providers’ adoption to understand overall team adoption). However, in some studies, these distinctions between the units of analysis and observation were not clearly drawn, explained, or appropriate. For instance, in a study examining practitioners participating in a quality improvement initiative, the study team assessed group level sustainability by asking individual practitioners to discuss their perceptions of sustainability in interviews. It was not clear how the research team arrived at their conclusions about group level sustainability from individual reports, which limits transparency and replicability. Furthermore, the lack of clarity around units of observation and analysis muddles the causal pathways that we are trying to understand in implementations outcomes research because mechanisms of change may differ among individual, group, organizational, and system levels.

A related issue involved limited explanation as to why units of observations (e.g., individual’s perceptions of appropriateness) can and should be aggregated to reflect higher levels in the analysis (e.g., organizational level appropriateness). Aggregating individual level data to the group, team, or organizational level requires a strong theoretical justification that bottom-up processes exist to create a shared characteristic [29]. In the example, sufficient theory was needed to demonstrate that appropriateness was an organizational level construct (unit of analysis) and reflected a shared perception of appropriateness among individuals (unit of observation). This type of study also requires an analytic design that allows the researcher to rigorously test this assumption [29], including sufficient sample sizes to account for between-group effects [30, 31]. During our data charting process, lack of clarity in how unit of observation and unit of analysis were distinguished and treated, and why, made it difficult to assess the presence of such considerations.

In response, *our sixth recommendation is to state the unit of analysis and unit of observation for each implementation outcome.* Observations may be generated by individual clients/patients, individual providers, teams, organizations, or another type of system. However, these observations may be aggregated in some way to reflect an implementation outcome at a higher level (e.g., assessing team adoption based on an aggregation of each individual member’s adoption). We urge the reader to ensure that the level of analysis theoretically and methodologically aligns with who provided data and the level of data collection described in in “Recommendation 4: data source and measurement” section. If conducting multilevel analyses and the units of observation and analysis are different, we also encourage the reader to include theoretical and analytical justification when aggregating implementation outcome data to a higher level of analysis [29].

## Discussion

### Why do we need another set of recommendations for implementation research?

First, we believe that as the field of implementation science grows and matures, researchers should continually hold their work to higher reporting standards so that they can delve deeper in and contribute more specifically to the field. This is especially important because implementation outcomes research often involves multilevel transactional relationships and processes. Second, this paper was not an abstract theoretical exercise. The development of the proposed recommendations—and the realization of the need to do so—was directly informed by our hands-on experience conducting a scoping review [25]. Third, our recommendations can be used to elaborate upon outcomes-related sections of existing guidelines. For example, our recommendations can be layered onto StaRI components that mention implementation outcomes (checklist item #2 for the “Abstract” section) and outcomes more broadly (checklist items #11 and #12 for the evaluation components of “Methods” section and checklist item #18 for the “Results” section) [14]. Fourth, our recommendations provide a template for reviewers and editors who want to offer suggestions for improving the study’s contribution to the implementation outcomes knowledge base. Finally, and most importantly, the current lack of implementation outcomes reporting guidelines negatively affects the usability, rigor, and impact of implementation outcomes research.

### What are potential challenges of using these recommendations?

First, individuals who conduct implementation outcomes research represent a wide range of professional backgrounds, research training, familiarity with terms, disciplinary standards of rigor, and study contexts. Elements of our recommendations may be more difficult to put into practice depending on the researcher’s background and the nature of “the thing” being assessed. Second, many studies that could contribute to the field of implementation science are designed to practically assist a specific population, rather than to intentionally generalize results for the sake of building the science. Third, following these reporting recommendations could lead to more content and challenges managing word and space limitations.

## Conclusion

Our scoping review experience illustrated why implementation outcomes research is difficult to conduct, report—and perhaps—even more difficult to consolidate across studies. There are multiple moving parts that researchers may have to juggle, including more than one referent, multiple stakeholder groups providing data, multiple levels of analysis, and varying rates of observable change over the course of the implementation process. This paper advances the 2011 implementation outcomes taxonomy and research agenda in two ways. First, we bring the 2011 research agenda to life with concrete examples drawn from a wide swath of existing literature. For example, the 2011 research agenda drew our attention to the importance of the consistency of implementation outcomes terminology [3]. In this paper, we illustrate three specific ways that this can show up in the literature if not appropriately addressed. This makes it easier for implementation researchers to both identify and avoid these issues. Our examples also reflect how the field has changed over the last 10 years. For example, the 2011 research agenda drew our attention to the importance of specifying the referent for rating the outcome [3]. With the growth of implementation strategy research and hybrid designs in recent years, our examples allowed us to show how messy—and how important—clear referent specification can be in implementation outcomes research. The second way that we advance implementation outcomes research is by offering solutions. We provide six pragmatic recommendations for improved reporting. These are accompanied by an easy-to-use audit worksheet and a list of exemplar articles that researchers, funders, and reviewers can refer to when designing, conducting, and assessing implementation outcomes studies.

## Supplementary Information

**Additional file 1: Table 1.** Audit worksheet organized by manuscript or grant proposal section.

**Additional file 2: Table 2.** Exemplar articles by recommendation.

## Data Availability

Not applicable.
