# Supplementary material for: Six practical recommendations for improved implementation outcomes reporting
Source: Implement Sci. 2022 Feb 8;17:16. doi: 10.1186/s13012-021-01183-3 (PMC8822722; doi:10.1186/s13012-021-01183-3)
Supplement: Supplementary file 2 — Additional file 2: Table 2. Exemplar articles by recommendation. [file 13012_2021_1183_MOESM2_ESM.docx]

Additional file 2.

*Exemplar articles by recommendation.*

| **Recommendation 1: Clearly state each implementation outcome and provide an operational definition that the study will use. Ensure consistent use of outcomes terms and operational definitions across manuscript sections and provide an explanation if using the taxonomy in a new way or merging terms.**   - Audit Worksheet sections: Introduction - item #2; Methods - item #1 | | |
| --- | --- | --- |
| Citation | Reporting details that others can replicate | Example from article |
| Calo WA, Shah PD, Gilkey MB, Vanderpool RC, Barden S, Doucette WR, et al. Implementing pharmacy-located HPV vaccination: findings from pilot projects in five US states. Human Vaccines & Immunotherapeutics. 2019; 15(7-8): 1831-1838. | Authors provided a table with implementation outcome construct, implementation outcome definition, and example measure from the study. | See table on page 1832. Example: construct (service penetration), definition (extent to which an evidence-based practice is integrated within a service system and its subsystems; also known as “reach”), example measure (number of HPV vaccine doses given to eligible patients contacted during the pilot project). |
| Cawthon C, Mion LC, Willens DE, Roumie CL, Kripalani S. Implementing routine health literacy assessment in hospital and primary care patients. The Joint Commission Journal on Quality and Patient Safety. 2014;40(2):68-AP1. | Authors defined outcome and described assessment in a single sentence. | See page 72. Example: Acceptability—the perception among stakeholders that a given practice is agreeable or satisfactory—was assessed qualitatively from administrators and frontline nursing personnel. |
| **Recommendation 2: Specify how each implementation outcome will be or was analyzed relative to other constructs.**   - Audit Worksheet sections: Introduction - item #4; Methods - item #3 | | |
| Citation | Reporting details that others can replicate | Example from article |
| Brookman-Frazee L, Zhan C, Stadnick N, Sommerfeld D, Roesch S, Aarons GA, et al. Using survival analysis to understand patterns of sustainment within a system-driven implementation of multiple evidence-based practices for children’s mental health services. Frontiers in Public Health. 2018; 6:54. | Authors described an analytic plan in which sustainment was the dependent variable. | See page 4: “A multivariate Cox regression (semi-parametric survival analyses) model was performed to determine the unique contribution of each predictor variable to the sustainment of therapists’ overall EBP practice delivery. The Cox regression model was selected because, as a semi-parametric model, no assumption had to be made about the distribution of the survival time. Survival time represented the time elapsed, in units of months, from the time of the therapist’s first claim to the time of the therapist’s final claim for any of the six EBPs. The binary outcome variable was (1) sustained delivery (i.e., censored) vs. (2) discontinued delivery (i.e., failure event).” |
| Williams NJ, Glisson C, Hemmelgarn A, Green P. Mechanisms of change in the ARC organizational strategy: Increasing mental health clinicians’ EBP adoption through improved organizational culture and capacity. Administration and Policy in Mental Health and Mental Health Services Research. 2017;44(2):269-83. | Authors provided a figure that showed where adoption fits into the hypothesized analytic model as a dependent variable. They also specified adoption in the data analysis narrative. | See figure on page 271. See page 274: “Analyses were implemented using the TWOLEVEL procedure in Mplus software (Version 7). Models with a dichotomous outcome (i.e., EBP adoption) incorporated a logit link function (i.e., two level mixed effects logistic regression).” |
| **Recommendation 3: Specify “the thing” that each implementation outcome will be measured in relation to.**   - Audit Worksheet sections: Introduction - item #5; Methods - items #5 and #6; Results - items #2 and #3; Discussion - item #2 | | |
| Citation | Reporting details that others can replicate | Example from article |
| Ware P, Ross HJ, Cafazzo JA, Laporte A, Gordon K, Seto E. Evaluating the implementation of a mobile phone–based telemonitoring program: longitudinal study guided by the consolidated framework for implementation research. JMIR mHealth and uHealth. 2018;6(7): e10768. | Authors specified how each outcome will be measured in a section of the paper titled “Implementation Outcomes” and provided a table that outlined the indicators for each implementation outcome. | See page 3: “We selected 4 implementation outcomes from Proctor et al.’s Implementation Outcomes Framework as measures of the implementation success. In addition, data on the outcomes, defined below, were collected after 4 and 12 months of the launch through a document review process and semi-structured interviews. Example: Adoption: The number of clinicians deciding to monitor patients using the Medly system. See Table 2 on page 4. |
| Haine-Schlagel R, Brookman-Frazee L, Janis B, Gordon J. Evaluating a learning collaborative to implement evidence-informed engagement strategies in community-based services for young children. Child & Youth Care Forum. 2013; 42: 457-473. | Authors described that the Modified Practice Attitudes Scale was used to assess participant attitudes towards EBPs, which is listed under measures of acceptability. | See page 463: “At the start of the initial LC learning session, participants completed a Pre-LC Survey and a measure on attitudes about EBPs. A total of 26 of 29 participants completed these assessments. At the end of the LC, participants completed an LC Perceptions Survey as well as the attitudes measure again. A total of 22 of 27 participants completed these assessments. Both surveys were developed to evaluate this LC.” |
| Jackson CB, Herschell AD, Schaffner KF, Turiano NA, McNeil CB. Training community-based clinicians in parent-child interaction therapy: The interaction between expert consultation and caseload. Professional Psychology: Research and Practice. 2017; 48(6): 481-489. | Authors specified the referent for the implementation outcomes in the data analysis section. | See page 484: “To examine whether consultation call attendance was associated with clinician outcomes (knowledge change, skill) and implementation outcomes (acceptability, feasibility), we estimated a series of regression analyses for the four separate outcomes.” |
| **Recommendation 4: Report who provided data and the level at which data were collected for each implementation outcome, and what type of data was or will be collected and used to assess each implementation outcome.**   - Audit Worksheet sections: Methods - items #7, #9, #10 | | |
| Citation | Reporting details that others can replicate | Example from article |
| Ober AJ, Watkins KE, Hunter SB, Ewing B, Lamp K, Lind M, et al. Assessing and improving organizational readiness to implement substance use disorder treatment in primary care: findings from the SUMMIT study. BMC family practice. 2017;18(1):1-13. | Authors specified who provided the data and when. | See pages 5-6: “For the present analysis, we used data from staff surveys conducted before the organizational readiness intervention was executed and 18-months later (and prior to the RCT) to assess changes in acceptability, perceptions of appropriateness and feasibility of, and intention to adopt SUD treatment during this period.” |
| Reynolds SS, McLennon SM, Ebright PR, Murray LL, Bakas T. Program evaluation of neuroscience competency programs to implement evidence‐based practices. Journal of evaluation in clinical practice. 2017;23(1):149-55. | Authors specified who provided the data and how. | See page 151: “In the fall of 2015, a qualitative study using one‐on‐one interviews with neurocritical care nurses was completed to evaluate the Stroke and Spinal Cord Injury Competency Programs.” Table 1 of the manuscript on page 151 includes the semi-structured interview questions with the implementation outcome they are meant to capture in parentheses at the end of the questions.” |
| **Recommendation 5: State the number of time points and the frequency at which each outcome was or will be measured.**   - Audit Worksheet section: Methods – items #13 and #14 | | |
| Citation | Reporting details that others can replicate | Example from article |
| Domitrovich CE, Li Y, Mathis ET, Greenberg MT. Individual and organizational factors associated with teacher self-reported implementation of the PATHS curriculum. Journal of school psychology. 2019; 76:168-85. | Authors stated the timing and frequency of survey administration to participants. | See page 174: “Teachers who were trained to deliver PATHS were asked to complete a self-report survey twice a year in the Winter (late January-early February) and Spring (late April-early May) A baseline administration of the survey also took place in the fall of the first year. This survey included questions that were innovation-specific such as teachers' attitudes towards PATHS, their professional experience, and their perceptions of the administration's support for the program. At each time point, the survey also asked teachers to provide self-report ratings of their PATHS implementation. Of the 299 teachers that received PATHS training, 235 (78.6%) responded to the baseline self-report survey, 249 (83.3%) completed the winter 2015 survey, and 250 (83.6%) completed the spring 2015 survey. In Year 2, 159 K-2 teachers took the winter 2016 survey (53.2%) and 82 took the spring 2016 survey (27.4%).” |
| Lindholm LH, Koivukangas A, Lassila A, Kampman O. What is important for the sustained implementation of evidence-based brief psychotherapy interventions in psychiatric care? A quantitative evaluation of a real-world programme. Nordic journal of psychiatry. 2019;73(3):185-94. | Authors stated the number of survey timepoints and the duration between each. | See page 187: “Three survey points (q1, q2, q3) were set to obtain longitudinal data. The first two questionnaires were administrated in refresher seminars: q1 one year after the initial training and q2 a year after q1. The final questionnaire, q3, came three years after q2 (i.e. 4–5 months after the programme closed) and was administered at a normal weekly meeting of each unit.” |
| Sigmarsdóttir M, Forgatch MS, Guðmundsdóttir EV, Thorlacius Ö, Svendsen GT, Tjaden J, et al. Implementing an evidence-based intervention for children in Europe: Evaluating the full-transfer approach. Journal of Clinical Child & Adolescent Psychology. 2019;48(sup1): S312-S25. | Using data extracted from databases, the authors stated the number of “generations” of data available from each country involved in the study. | See page S318: “Reliable FIMP coders rated the certification sessions, which for each therapist consisted of four video-recorded sessions of individual family therapy. Data were available for six generations in Iceland, eight in Denmark, and four in the Netherlands. Information about the number of therapists who started, completed, and their duration of practice in the program was gathered from national databases for the programs and yearly reports.” |
| **Recommendation 6: State the unit of analysis and unit of observation for each implementation outcome.**   - Audit Worksheet section: Methods - items #9, #10, #11 | | |
| Citation | Reporting details that others can replicate | Example from article |
| Brunk MA, Chapman JE, Schoenwald SK. Defining and evaluating fidelity at the program level in psychosocial treatments. Zeitschrift für Psychologie. 2014; 222(1): 22-29. | Authors explicitly defined the unit of observation for program implementation. | See page 23: “For the purposes of this study, program implementation was defined as implementation by MST teams. In the case of MST, a ‘‘program’’ is, at minimum, a single team of 2–4 therapists and a clinical supervisor.” |
| Bruns EJ, Parker EM, Hensley S, Pullmann MD, Benjamin PH, Lyon AR, et al. The role of the outer setting in implementation: associations between state demographic, fiscal, and policy factors and use of evidence-based treatments in mental healthcare. Implementation Science. 2019;14(1):1-13. | Authors clearly articulated the unit of observation for EBT adoption at the state level. | See page 4: “State-level adoption of behavioral health EBTs included six EBTs tracked by the NRI surveys over the study period.” |
| Johnson JE, Hailemariam M, Zlotnick C, Richie F, Sinclair J, Chuong A, et al. Mixed Methods Analysis of Implementation of Interpersonal Psychotherapy (IPT) for Major Depressive Disorder in Prisons in a Hybrid Type I Randomized Trial. Administration and Policy in Mental Health and Mental Health Services Research. 2020;47(3):410-26. | Authors explained the difference between the unit of observation (patient and provider/administrator) and unit of analysis (prison system). | See pages 413-414. Example for unit of observation: “Acceptability, relative advantage, relative priority, and compatibility of IPT to prison providers and administrators were assessed using the Stakeholder Acceptability Survey (SAS).” Unit of analysis: “Quantitative measures were analyzed descriptively and interpreted in terms of CFIR constructs that may be facilitators and barriers of IPT implementation in the two prison systems.” |
